# Mapping lung cancer ventilation dynamics using functional imaging and lung mechanics

**DOI:** 10.1242/dmm.052559

**Published:** 2025-12-29

**Authors:** Ronan Smith, Nicole Reyne, Daniel Batey, Nina Eikelis, Marie-Liesse Asselin-Labat, Martin Donnelley

**Affiliations:** ^1^ Robinson Research Institute, University of Adelaide, Adelaide 5006, Australia; ^2^ Adelaide Medical School, University of Adelaide, Adelaide 5000, Australia; ^3^ Respiratory and Sleep Medicine, Women‘s and Children‘s Hospital, Adelaide 5006, Australia; ^4^ Personalised Oncology Division, Walter and Eliza Hall Institute of Medical Research, Melbourne 3052, Australia; ^5^ Department of Medical Biology, The University of Melbourne, Melbourne 3052, Australia; ^6^ 4DMedical, Victoria 3053, Australia

**Keywords:** Lung cancer, Animal models, Lung disease, Lung function, X-ray velocimetry, Permetium, flexiVent

## Abstract

*In vivo* models that replicate and reproduce human lung cancer and its response to therapy are necessary for the development of new therapeutic strategies and understanding drug resistance. Imaging lung tumors in live animals to monitor tumor growth and response to therapy is challenging owing to the location of the lungs and their constant movement during breathing. X-ray velocimetry (XV) is a novel functional lung imaging technique that maps regional lung expansion during breathing, providing spatial information on where ventilation changes occur. The aim of this pilot study was to use XV and flexiVent lung mechanics assessments to determine the effect of tumor growth on lung function in mice at 2 or 3 weeks post tumor induction and to evaluate the efficacy of these two tools. Histological analysis showed that tumor growth was not uniform between animals. At 3 weeks post tumor induction, some XV ventilation and flexiVent lung mechanics parameters were significantly different from baseline. Both techniques gave metrics that correlated with the tumor counts from the histology. In some mice, XV revealed localized regions with altered expansion rates.

## Teaser

**Summary:** We demonstrate that X-ray velocimetry imaging can be used to track tumor burden through changes to ventilation in a mouse model of lung cancer.

## INTRODUCTION

Lung cancer remains the leading cause of cancer-related deaths worldwide, with a 5-year survival rate of less than 20% (Bray et al., 2024), primarily due to late diagnosis and limited treatment options. Non-small cell lung cancer (NSCLC), which accounts for ∼85% of lung cancer cases, frequently harbors genetic mutations that drive tumor growth and progression (Chevallier et al., 2021). In lung adenocarcinoma (LUAD), the most common subtype of NSCLC, the most common oncogenic driver mutations are mutations in *KRAS* (∼30% of LUAD cases) and loss-of-function mutations in the tumor suppressor gene *TP53* [∼50% of LUAD cases (Cancer Genome Atlas Research Network, 2014)]. Although activation of the *KRAS* oncogene in murine lungs induces lung adenoma formation (Tuveson et al., 2004), co-occurring *TP53* mutation accelerates tumor onset and the invasive properties of the tumors, leading to LUAD formation (Muzumdar et al., 2016).

Mouse models that recapitulate the genetic and pathological features of human LUAD have been extensively used to study lung cancer initiation, progression and response to therapy. Cell lines established from the *KRAS^G12D^/TP53^mut^* animal models (Weeden et al., 2023) further allow for the establishment of lung tumors in a controlled and reproducible manner, enabling tumor growth dynamics, immune responses and potential therapeutic interventions to be studied *in vivo*. One of the major challenges in the use of animal models of lung cancer is monitoring tumor burden and its impact on respiratory function in live animals.

Current methods to assess tumor burden *in vivo* in preclinical models utilize X-ray computed tomography (microCT) (Zaw Thin et al., 2022) or require the use of luciferase-expressing cells for bioluminescence imaging (Madero-Visbal et al., 2012). Although microCT provides an accurate overview of tumor location, distinguishing tumor from normal tissue can be difficult without contrast agents. Bioluminescence can be sensitive and easy to use; however, signal attenuation varies depending on how deep the tumors are in the thorax, reducing quantitative accuracy and precise information on location. In addition, both methods do not provide information on the impact of tumor burden on lung health and respiratory function.

The flexiVent system is the current gold standard for assessing lung function in small-animal models. The flexiVent applies the forced oscillation technique to provide precise measurements of airway resistance, lung compliance and elastance, offering detailed insights into how tumors alter pulmonary mechanics. In addition, negative pressure forced expiration (NPFE) testing can be used to assess airflow in a manner analogous to human spirometry. As lung tumors develop, they would be expected to cause airway obstruction, reduced compliance, impaired gas exchange and altered airflow, which the flexiVent should be able to quantify. To date, there have been no reports of the flexiVent being used to characterize changes in lung mechanics from lung cancer.

X-ray velocimetry (XV) functional lung imaging has emerged as a powerful, non-invasive technique for assessing regional ventilation in preclinical models (Asosingh et al., 2022; Reyne et al., 2024a,b, 2025; Stahr et al., 2016). XV imaging allows real-time visualization of lung motion and airflow distribution, capturing subtle changes in regional lung mechanics and ventilation heterogeneity. There are no reports of XV functional lung imaging being applied to lung cancer. However, XV could be particularly valuable for evaluating how lung tumor development affects localized airflow and how interventions may restore normal ventilation patterns. Unlike traditional lung function tests, which provide global measurements, XV imaging provides regional insights into tissue motion and lung ventilation, potentially enabling a more comprehensive understanding of tumor-induced lung dysfunction.

This pilot study examined a previously established mouse model of lung cancer (Weeden et al., 2023) using flexiVent lung function testing and XV imaging. The aims were to begin to assess the timing of lung cancer progression and to evaluate the efficacy of these tools in a cancer model. We hypothesized that these methods could provide critical insights into tumor biology and lung mechanics.

## RESULTS

Data were collected from control mice (*n*=2) and lung tumor-bearing mice 2 weeks (*n*=3) or 3 weeks (*n*=3) after tumor cell injection. The mice were the same age, and there was no significant difference in the weight of the three groups. All mice tolerated the tumor induction and XV imaging. Lung mechanics could not be assessed by flexiVent in one of the 3-week animals (referred to as C1) due to a system leak.

### XV imaging

**Fig. 1.**
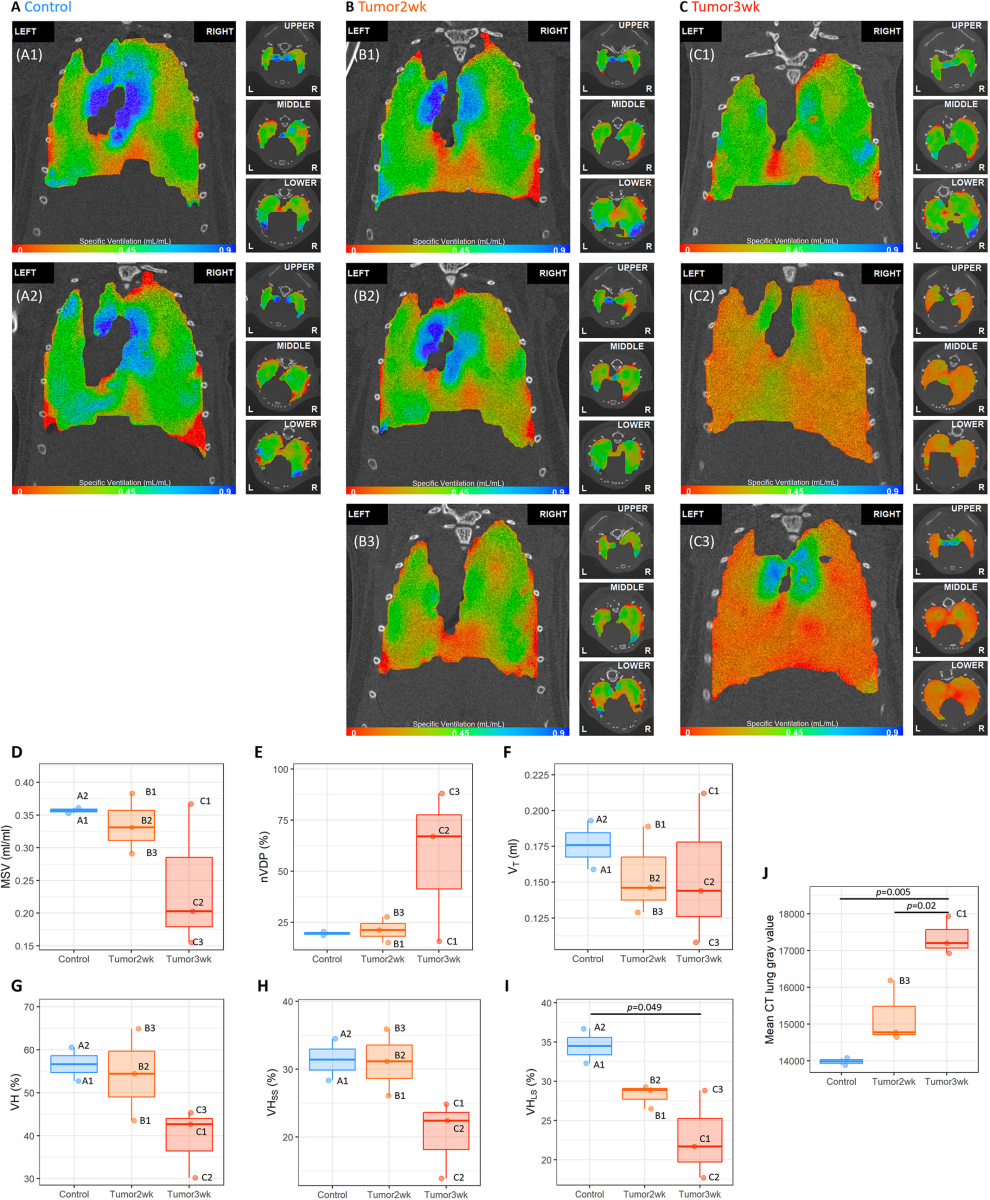
**X-ray velocimetry (XV) ventilation maps.** (A-C) Coronal and axial slices from the 3D ventilation maps produced by XV from control mice (A), the 2-week group (B) and the 3-week group (C). Green indicates average ventilation, red indicates below average ventilation, and blue indicates above average ventilation. (D-I) Global XV parameters – mean specific ventilation (MSV; D), normalized ventilation defect percentage (nVDP; E), tidal volume (V_T_; F), ventilation heterogeneity (VH; G), small-scale VH (VH_SS_; H) and large-scale VH (VH_LS_; I) – were variable, but only VH_LS_ was significantly lower in the 3-week group than in control. (J) The mean computed tomography (CT) gray value was significantly lower in the 3-three week group than in control. Note that the individual mice are labeled to enable their characteristics to be tracked across the datasets. Boxplots show the median and interquartile range (IQR); whiskers show 1.5×IQR.

XV produces a three-dimensional map of the specific ventilation at ∼6000 voxels (0.4 mm×0.4 mm×0.4 mm in size) across the lung. The 3-week group exhibited much more obvious reductions in specific ventilation (red regions in Fig. 1A-C) across the entire lung than the control or 2-week groups (Fig. 1A-C). Large-scale ventilation heterogeneity (VH_LS_) was significantly reduced in the 3-week group compared to that in the control, but there were no statistically significant differences in the mean specific ventilation (MSV), normalized ventilation defect percentage (nVDP), tidal volume (V_T_) or ventilation heterogeneity (VH) between the control and tumor-bearing mice at either 2 or 3 weeks post tumor induction (Fig. 1D-I).

**Fig. 2.**
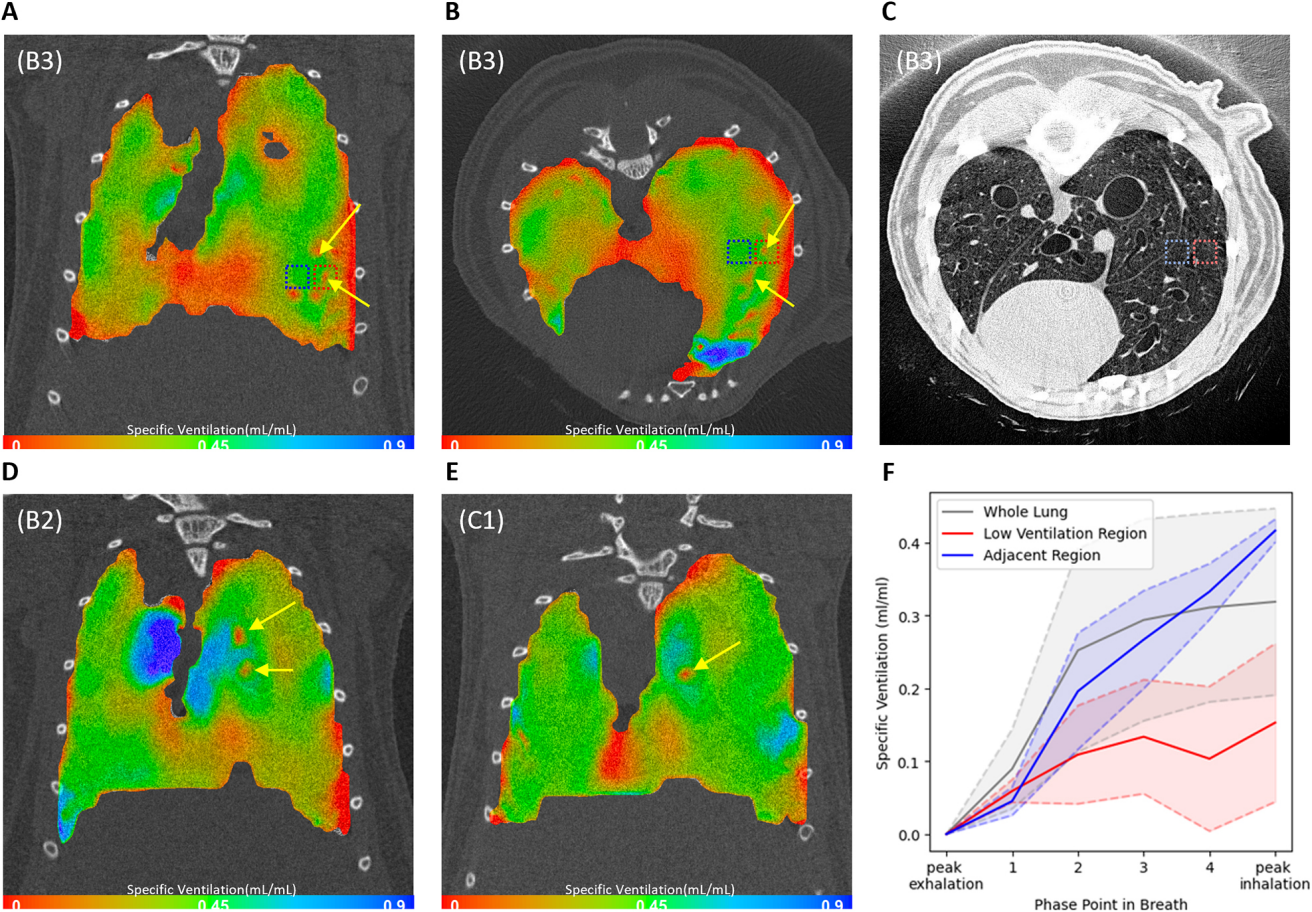
**Case studies of XV ventilation maps showing localized areas of very low ventilation.** (A-C) Multiple small regions of low ventilation (yellow arrows) were seen in the coronal (A) and axial (B) slices from mouse B3 from the 2-week group, although no obvious change was visible in the post-mortem CT slice from the corresponding area (C) (note that the lungs moved between the XV and post-mortem CT scans because the animal was removed from the scanner for flexiVent testing, so the images do not correlate perfectly). (D,E) Similar regions (yellow arrows) were identified in mouse B2 (D) from the 2-week group and in the least severely affected mouse C1 (E) from the 3-week group. Green indicates average ventilation, red indicates below average ventilation, and blue indicates above average ventilation. (F) Specific ventilation across the five phase points throughout the breath for mouse B3 shown in A-C. The gray line is the inflation of the whole lung, the red line is the inflation in one of the areas of interest (red dotted line box in A,B), and the blue line is the inflation in a nearby region of normal ventilation (blue dotted line box in A,B). The shaded areas are the standard deviations in the respective regions.

Further inspection of the ventilation maps showed that some of the mice had small, localized regions of low ventilation (shown by the yellow arrows in Fig. 2). These regions could be seen in two of the 2-week mice (mice B2 and B3 in Fig. 2A,B), with no obvious structural changes visible in these areas in the post-mortem CT scan (Fig. 2D). Similar localized low ventilation regions could also be seen in a 3-week mouse (C1 in Fig. 2C) that did not have the large clear low ventilation regions seen in Fig. 1.

Further analysis was performed to investigate these small, localized ventilation defects. We defined a region of interest of ∼0.8×0.8×0.8 mm surrounding one of these regions (the position is shown by the red dotted line box in Fig. 2A). The MSV during the breath in this region of interest was compared to an adjacent identically sized region (blue dotted line box) to explore any temporal changes. Curves showing the expansion of these regions throughout inspiration compared to the expansion of the whole lung are shown in Fig. 2F, with the shaded areas showing the standard deviation of specific ventilation within these regions. The inflation of the whole lung was non-linear, with the lung inflating much more rapidly at the beginning of the breath, before plateauing. A similar pattern to this was seen in the region adjacent to the region of interest. However, within the region of interest, the inflation was very different, slowly expanding across the breath and remaining much lower than in the rest of the lung.

The computed tomography (CT) scans showed that the tumors were more dense than the normal lung tissue, with the mean CT gray level increasing significantly at the 3-week time point (Fig. 1J).

### flexiVent respiratory mechanics

**Fig. 3.**
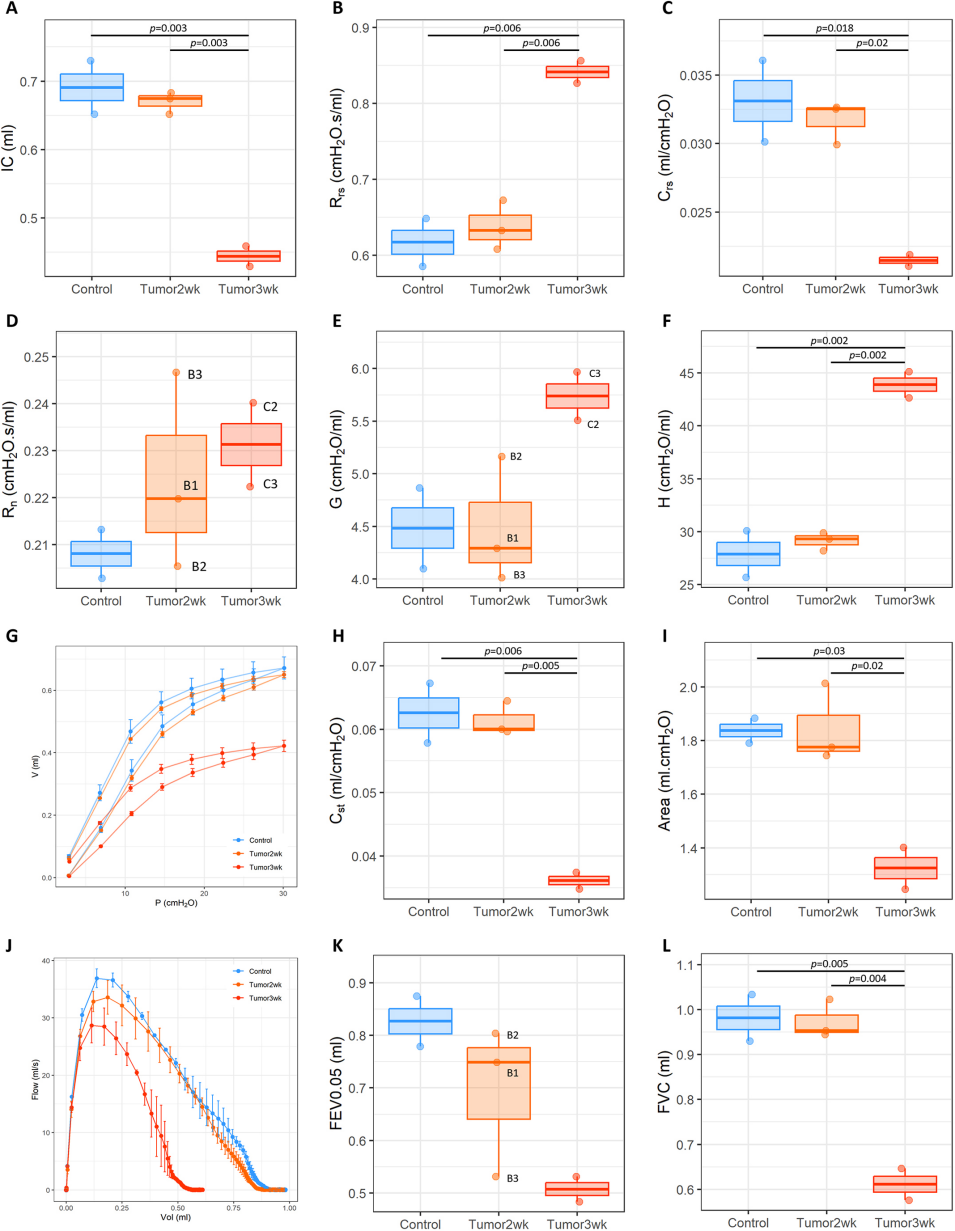
**Lung mechanics results.** The mechanics of the respiratory system are significantly altered at 3 weeks but not 2 weeks post tumor induction. (A) Inspiratory capacity (IC) from the deep inflation maneuver. (B,C) Respiratory system resistance (R_rs_; B) and compliance (C_rs_; C) from the single compartment model. (D-F) Central airway resistance (R_n_; D), tissue damping (G; E) and tissue elastance (H; F) from the forced oscillation technique. (G-I) The stepwise pressure–volume (PV) loop (G), static compliance (C_st_; H) and PV loop area. (J-L) Flow–volume plot (J) and flow parameters [forced expiratory volume in 0.05 s (FEV0.05; K) and forced vital capacity (FVC; L)] from the negative pressure forced expiration test. Boxplots show the median and IQR; whiskers show 1.5×IQR.

Selected results from the flexiVent testing are shown in Fig. 3. There were no significant differences between the control and 2-week tumor groups for any of the tests. However, at 3 weeks, mice exhibited significant reductions in inspiratory capacity (IC) and respiratory system compliance (C_rs_), along with an increase in respiratory system resistance (R_rs_). Tissue elastance (H) was significantly increased at 3 weeks. Average pressure–volume (PV) loops constructed from mean data showed a downward shift in the PV relationship of the 3-week mice, with corresponding reductions in static compliance (C_st_) and PV loop area. The NPFE test showed altered flow characteristics and a reduced forced vital capacity (FVC) at 3 weeks.

### Lung tissue histology

**Fig. 4.**
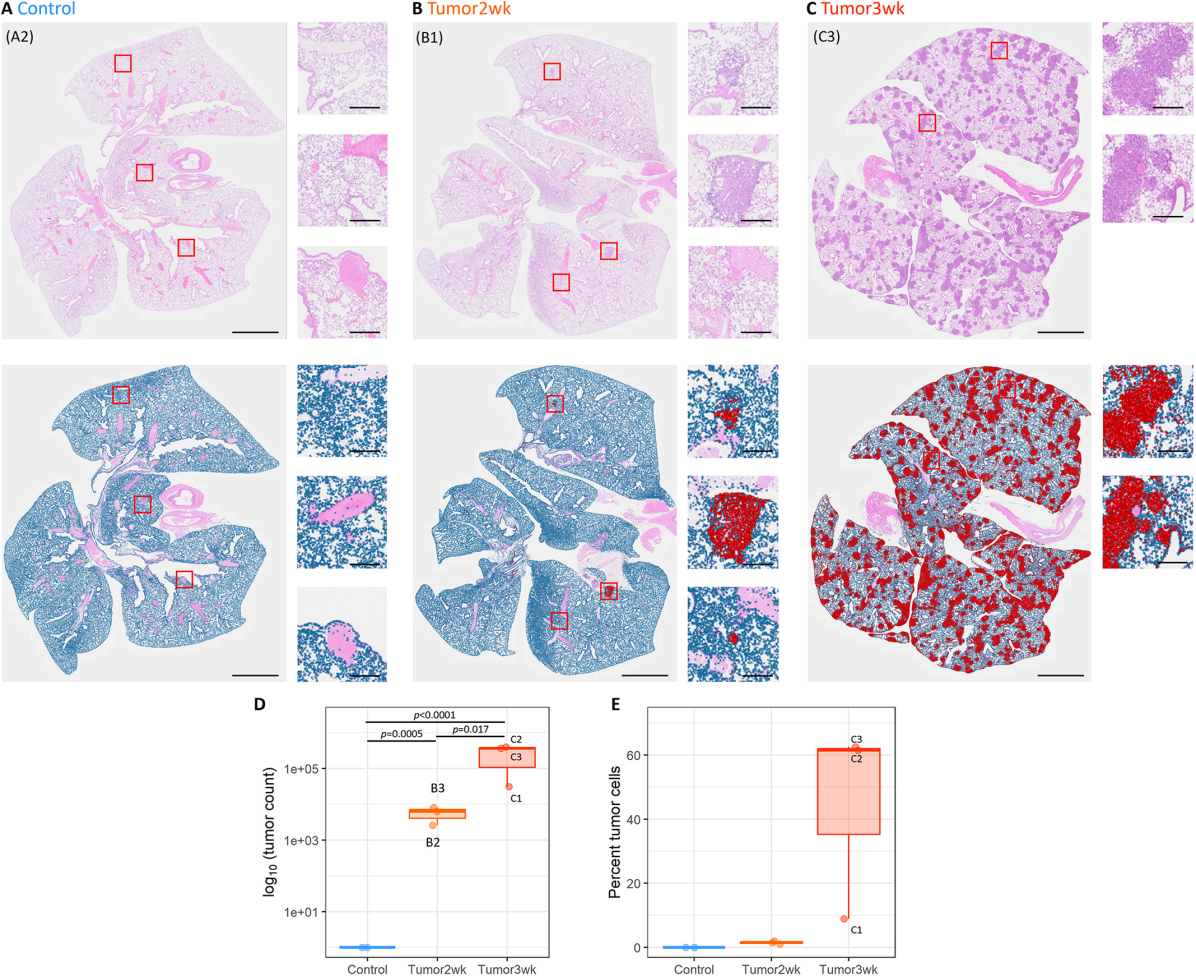
**Histological images of tumor-containing lung tissue.** (A-C) Representative images of lung sections from control (A), 2-week tumor (B) and 3-week tumor (C) mice stained with Hematoxylin and Eosin (top row) and after classification of cells as normal (blue) or tumor (red) (bottom row). Scale bars: 2 mm, 200 μm (insets). (D,E) Tumors were quantified and represented as total tumor counts (D) and tumor burden (E) as a percentage of total lung area. Boxplots show the median and IQR; whiskers show 1.5×IQR.

**Fig. 5.**
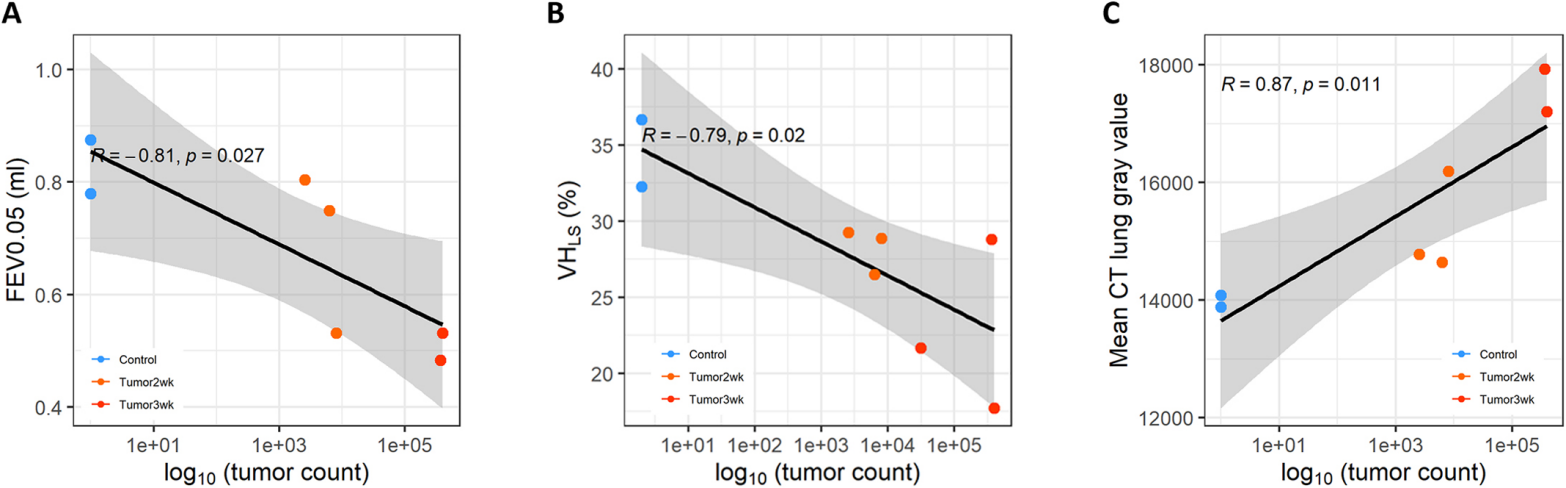
**Parameter correlations.** (A-C) Statistically significant correlations between tumor burden and the flexiVent parameter FEV0.05 (A), XV parameter VH_LS_ (B) and average CT gray value (C). There were no other significant correlations between the tumor burden and any other parameter.

Histological analysis was performed on the excised lung tissue. Tissue sections from both control animals exhibited normal airway and alveolar structure. In contrast, in the two lung cancer groups, obvious tumors were identified across the lung, with tumor burden representing 1.5% of the lung area in the 2-week group and 44.3% in the 3-week group (Fig. 4). Mouse C1, which appeared to have relatively normal MSV, nVDP, V_T_ and VH, as measured by XV (Fig. 1), was the mouse that had the lowest tumor burden by histology. Correlations between log_10_(tumor count) and each of the XV and flexiVent parameters were assessed, with the relationships to forced expiratory volume in 0.05 s (FEV0.05), VH_LS_ and mean CT gray value found to be statistically significant (Fig. 5).

## DISCUSSION

The aim of this pilot study was to evaluate a previously established mouse model of lung cancer using flexiVent lung function testing and XV imaging to assess the timing and extent of lung cancer progression. XV ventilation maps (Fig. 1A-C) demonstrated a clear reduction in ventilation across the whole lung at 3 weeks post tumor induction, with more subtle alterations observed at the 2-week time point. Considering that tumor burden accounted for only 1.5% of the lung area in the 2-week group, the fact that there were measurable changes in ventilation suggests that XV could have high sensitivity in this application.

Interestingly, the severity of ventilation defects varied between animals. For example, one 3-week tumor-bearing mouse (C3) showed widespread ventilation defects, while another (C1) only exhibited mild changes. A similar pattern was also observed in the 2-week group, in which one animal (B1) appeared to be more mildly affected, displaying a normal ventilation map comparable to that of the control animals. This variability was reflected in the global metrics and in the tumor burden quantification. For example, C3 had a markedly reduced MSV and V_T_, whereas C1 exhibited relatively normal MSV and nVDP, but did show a reduction in VH. The only XV parameter that was significantly altered was the VH_LS_, specifically between the 3-week group and controls; however, this was likely to be caused by the small sample size and inter-animal variability.

Overall, tumor-bearing lungs tended to progress towards more homogeneous ventilation (reduced VH), coupled with overall lower airflow (reduced MSV and increased nVDP), suggesting that, overall, the tissue is stiffer. This matches the flexiVent findings in which the C_rs_ and C_st_ were significantly reduced at 3 weeks. However, it is likely that, as the tumors progress, VH increases locally owing to the patchy tumor tissue. For example, the 2-week animals in which we identified small areas of low ventilation (B2 and B3) had high small-scale ventilation heterogeneity (VH_SS_) values, which likely reflect this phenomenon. Over time, the lung motion overall becomes more homogeneous, resulting in reduced VH_LS_ at 3 weeks. In obstructive lung diseases, low VH indicates more homogeneous ventilation, which is typically associated with less patchy disease and preserved lung function (Reyne et al., 2025). Here, the reduced VH may still reflect homogeneous ventilation, but the overall level of ventilation was reduced (i.e. low MSV), indicating impaired lung function. Thus, both of these parameters must be considered together to interpret lung health.

The case study of the region of interest in mouse B3 (Fig. 2) adds to these findings. The whole lung ventilation, indicated by the gray line in Fig. 2, demonstrated a typical nonlinear inflation pattern, with rapid expansion occurring early in the breath, followed by a slower increase approaching peak inspiration. In contrast, the region of interest (red line in Fig. 2) showed an abnormal trajectory; rather than increasing in early inhalation, specific ventilation actually decreased between Phase 1 and 2. In obstructive diseases (Reyne et al., 2025) or models of obstruction (Reyne et al., 2024b), this altered specific ventilation trajectory would likely reflect mucus obstruction, resulting in delayed filling. However, in this model of lung cancer, we propose that this is the result of altered tissue properties (i.e. increased stiffness) caused by tumor growth that prevents normal lung tissue expansion. Interestingly, in this 2-week animal (B3), there were not substantial differences in tissue compliance; indeed, changes in compliance (C_rs_ and C_st_) did not become statistically significant until the 3-week timepoint. These findings suggest that XV imaging is sensitive enough to detect these subtle local functional impairments not evident in the global flexiVent lung mechanics testing or the structural CT imaging.

The flexiVent results indicated a significant decline in lung function at 3 weeks, but not at 2 weeks. At the 3-week timepoint, IC was significantly reduced, suggesting that the presence of tumors in the lung reduced the overall volume of air the mice were able to inhale. There was also a significant increase in overall R_rs_ and a decrease in C_rs_, indicating that tumors also impair the ability of the lungs to stretch and allow airflow during breathing. PV loops further supported these findings, showing a reduction in C_st_ and a smaller overall area under the curve, further indicating stiffer lungs with reduced IC. The NPFE maneuver revealed a significant decrease in FVC, consistent with reduced lung volume. Other parameters [e.g. Newtonian resistance (R_n_), tissue damping (G), FEV0.05] were not statistically different from control; but, as for the XV parameters, this was likely to be caused by the small sample size.

The histological findings confirmed the XV and flexiVent results, with tumor burden substantially increasing between 2 weeks and 3 weeks. Interestingly, we found strong correlations between the log_10_(tumor count) and the FEV0.05, VH_LS_ and mean CT gray value, but not with any of the other parameters.

### Limitations and outlook

The primary limitation of the model is the small sample sizes that we utilized in our pilot design. We expect that, with larger sample sizes, many of the parameter changes would become statistically significant, potentially at the 2-week timepoint. Importantly, however, we were still able to draw strong conclusions about the capabilities of XV imaging and establish the expected changes in flexiVent mechanics in this pilot study.

As this was a proof-of-concept pilot study, feature matching between the histology, CT and XV images was not performed. As discussed by Nolte et al. (2022), this is a challenging procedure and something that could be investigated in future studies (Nolte et al., 2022).

Although this mouse model recapitulates the characteristic of the human tumors at the molecular and cellular level, human tumors are predominantly one single tumor focus in a lung lobe, instead of the many lung tumor foci observed in all the lobes in this mouse model (caused by systemic delivery of the cancer cells). The large overall tumor burden in the mice would impact lung function in the whole volume of the lung, whereas in humans, tumors would affect a more restricted region of the lung. This suggests that there is likely to be significant value from moving toward human clinical trials rather than performing larger studies in mouse models. Nonetheless, that approach also presents challenges as human lung cancer patients often have comorbidities such as chronic obstructive pulmonary disease or fibrosis that complicate assessments.

Future longitudinal rodent studies could be used to track tumor progression in individual animals and enable improved evaluation of chemotherapeutics. The XV and CT scans are not necessarily terminal procedures. In this study, animals were only euthanized because a tracheostomy was required for successfully performing the selected flexiVent measurements. As we showed here, there can be significant differences in tumor growth rates, so longitudinal tracking of individual animals could reduce the total number of animals needed for studies and improve statistical power. However, the effect of any radiation from the repeated scanning on tumors would need to be understood.

As with all papers using XV imaging, this study refers to the data produced by XV as measuring ventilation; however, what is being measured is actually tissue expansion. In obstructive lung diseases, the elasticity of the tissue remains approximately constant, while the force being applied by air is reduced due to obstructed airways. This leads to a true reduction in ventilation, which is visible due to the lung reduced expansion of the lung tissue. However, in this model, the elasticity of the tissue has changed, and so tissue expansion and true ventilation are not analogous. Thus, further studies are required to fully understand how tissue properties such as stiffness affect the XV data and how this is altered by tumor growth.

### Clinical significance

In patients, tumor burden and response to therapy are primarily monitored by anatomical imaging (e.g. CT, magnetic resonance imaging) and nuclear medicine (e.g. positron emission tomography). These primarily assess the tumor’s size or metabolic activity, not its direct impact on lung function. These preliminary results suggest that combining anatomical imaging with quantitative, region-specific XV functional imaging could (1) provide earlier or more sensitive detection of clinically significant tumor progression; (2) help distinguish between stable disease on imaging but declining lung function (or vice versa); (3) support decision making for therapies or interventions (e.g. surgery versus continued chemotherapy).

Early functional changes detectable by XV may precede detectable anatomical abnormalities. Therefore, identifying subtle shifts in ventilation before significant structural changes appear on imaging could allow clinicians to identify patients at risk of rapid decline much earlier. If functional impairment – rather than just tumor size – is predictive of outcomes or complications such as respiratory failure, infection or tolerance to surgery, then XV functional imaging could be used to stratify patients for treatment risk or need for supportive care. XV imaging may provide value for guiding radiotherapy or surgical planning. Regionally specific impairment – as noted in our case study of mouse B3 – could guide surgeons or radiation oncologists to target or spare particular lung segments that are responsible for significant ventilation (e.g. Australian clinical trial ACTRN12612000775819).

### Conclusions

Using novel non-invasive *in vivo* XV lung function imaging in conjunction with gold-standard flexiVent whole-lung mechanics, we have performed a detailed study of lung function in a mouse model of lung cancer – the first of its kind. The results suggest that the severity of the model increases dramatically between weeks 2 and 3, with both the flexiVent and XV imaging detecting statistically significant changes between the control and 3-week groups, but not the 2-week group. Future human XV clinical trials, similar to those being performed for cystic fibrosis and other lung diseases, could reveal more about the alterations to airflow from lung cancer.

## MATERIALS AND METHODS

### Animals

All animal procedures were approved by the Walter and Eliza Hall Institute (2023.006) and South Australian Health and Medical Research Institute (SAM-23-065) Animal Ethics Committees. The parts of the Animal Research: Reporting of *In Vivo* Experiments (ARRIVE) guidelines that pertained to a pilot study were adhered to (Percie du Sert et al., 2020). All experiments were conducted in accordance with the National Health and Medical Research Council (NHMRC) Australian Code of Practice of the Care and Use of Animals for Scientific Purposes.

Lung cancer-bearing mice were supplied by the Walter and Eliza Hall Institute, and were generated by injection of a tumor cell line derived from a murine lung tumor that grew in a cre-inducible *Kras^G12D^*;p53^Δ/Δ^ C57BL/6 mouse model. Female age-matched mice received intravenous tail-vein injection of 1×10^5^ *Kras^G12D^*;p53^Δ/Δ^ lung cancer cells into C57BL/6 mice 2 or 3 weeks prior to the imaging, as previously described (Weeden et al., 2023). Mice were housed in a conventional facility with a 12-h light/dark cycle, and food and water were provided *ad libitum*.

### XV imaging

All XV imaging was performed as described previously (Asosingh et al., 2022; Reyne et al., 2025). Mice were anesthetized with an intraperitoneal injection of a mix of 1 mg/kg medetomidine (Ilium, Australia) and 75 mg/kg ketamine (Ceva, Australia). Once anesthetized, the mice were tracheostomized, and a short endotracheal tube (18 Ga BD Insyte plastic cannula bevel cut to 15 mm length) was inserted. Mice were then secured head high in a holder and placed onto the translation/rotation stage of a Permetium preclinical XV scanner (4DMedical, Australia) (Reyne et al., 2024a), configured with a sample to detector distance of 1700 mm. Mice were connected to a pressure-controlled small animal ventilator (4DMedical Accuvent 200) and ventilated at 150 breaths/min [200 ms inspiration (I) and 200 ms expiration (E); I:E ratio of 1:1] with a peak inspiratory pressure of 14 cmH_2_O and positive end-expiratory pressure (PEEP) of 2 cmH_2_O. An XV scan was acquired at a frame rate of 25 Hz, with ten phase points per breath and 600 projections per phase point. The X-rays were generated by a Rigaku MM09 X-ray source (40 kV, 2 mA) with a 40 μm molybdenum filter and detected by a Varex 2020DX flat-panel detector (1024×1024 pixels), with geometric magnification, giving an effective pixel size of 25.7 μm in the sample plane.

Specific ventilation volumetric data were produced by 4DMedical using their proprietary three-dimensional cross-correlation algorithm to quantify tissue displacement throughout the breath. Specific ventilation is defined as the change in volume of a voxel of the lung, divided by the volume of the voxel at the start of the breath. Unless explicitly stated, in this paper, any reference to XV refers to specific ventilation measured between peak exhalation and inspiration. XV ventilation maps can be viewed by taking two-dimensional slices in any plane. The slices shown here have anatomical features such as bone from the CT overlaid onto the ventilation data. Regions of interest in specific ventilation maps can be segmented in the same way as regions in CT, as we have demonstrated previously (Smith et al., 2025).

From the specific ventilation volume, MSV, ventilation defect percentage (VDP; the percentage of the lung with the specific ventilation below a threshold, typically 60% of the MSV), VH (the interquartile range divided by the mean, which gives a measure of the ventilation variance across the lung) and V_T_ were calculated. For this study, we report the nVDP, which was calculated using 60% of the MSV of the control population to define a threshold for defective regions, as described by Reyne et al. (2024b, 2025). VH was split into small scale (VH_SS_) and large scale (VH_LS_), showing heterogeneity across small (i.e. intra-lobe) and large (i.e. inter-lobe) spatial scales, respectively. These were calculated by applying high-pass and low-pass spatial filters before calculating VH.

### flexiVent respiratory mechanics

After XV scan acquisition, lung function assessments were performed using a flexiVent FX small-animal ventilator (SCIREQ, Montreal, Canada) as previously described (Reyne et al., 2025). To suppress spontaneous breathing during lung function measurements, the mice received an intramuscular dose of 0.6 mg/kg vecuronium bromide after being connected to the flexiVent. Briefly, the flexiVent was fitted with a FX2 mouse module and NPFE extension, and operated by flexiWare v8.0 software. Mice were connected to the flexiVent via a 18 Ga cannula, ventilated at a respiratory rate of 150 breaths/min, I:E ratio of 2:3, V_T_ of 10 ml/kg, and PEEP of 3 cmH_2_O. Lung mechanics measurements were made using a mouse mechanics scan script that was repeated three times – consisting of Deep Inflation (IC), SnapShot-150 (R_rs_, C_rs_), Quick-Prime 3 (R_n_, G, H), PVs-P (C_st_, curvature, area) and NPFE (FEV0.05, FVC, forced expiratory flow in 0.05 s) perturbations – with the Scireq automated algorithms set to default values (Reyne et al., 2025; Devos et al., 2017). For each parameter, three measurements were made per mouse and these were averaged. Data were excluded if the coefficient of determination was less than 0.9 for each model. After lung mechanics were assessed, the mice were humanely killed with an intraperitoneal injection of sodium pentobarbitone (∼200 mg/kg).

### CT

A CT volume can be reconstructed from the data acquired during the XV scan (referred to as the breathing CT); however, owing to respiratory and cardiac motion, the sharpness of this scan is limited. As the breathing CT and XV volumes align, the same segmentation was applied to the breathing CT. From this, the average CT gray values across the lung were calculated for each animal. To produce a sharper image of the lung structure, a second CT scan was performed after the flexiVent testing was performed, the animal was humanely killed and the heart had stopped beating (referred to as the post-mortem CT). That scan used 3000 projections, with all other Permetium parameters the same as in the XV scan described previously. The voxel size in both the reconstructed volumes was 25.7 μm.

### Histological analysis

The lungs were instillation fixed *in situ* under gravity at a constant fluid pressure of 25 cmH_2_O with 10% neutral buffered formalin (NBF). The lungs and heart were removed and suspended in 10% NBF overnight and then transferred into ethanol. The lungs were then embedded in paraffin. Tissue sections (5 µm) were cut from the formalin-fixed paraffin-embedded left lung blocks, and stained with Hematoxylin and Eosin. All slides were imaged in brightfield with an Olympus VS200 slide scanner, and images were analyzed with QuPath software (Bankhead et al., 2017). Cell segmentation and cell counting were performed in an automated manner using the QuPath Cell Detection algorithm applied to full-resolution images. Tumor burden was quantified in QuPath using Artificial Neural Network Multilayer Perceptron (ANN_MLP) object classifiers trained on manually annotated tumor and lung tissues.

### Statistics

All statistical analyses were performed in R version 4.4.1. Separate *t*-tests were used to determine whether the weight of the control and cancer mice was significantly different. For the XV and flexiVent analyses, the statistical findings were expressed in terms of estimated marginal means and confidence intervals returned from linear models fitted to the data. For every flexiVent and XV parameter, a standard linear regression model was fitted using the ‘lm’ function with a fixed effect of treatment. Post hoc pairwise comparisons for the fitted models were carried out using the emmeans package. The tumor count data were log_10_ transformed, the same linear regression model was applied, and the estimates and confidence intervals were assessed on the log scale. Owing to the small sample size, the results are graphically represented as individual data points and boxplots [median, box represents the interquartile range (IQR) and the whiskers 1.5×IQR] to show variability, and the relationships between the ventilation maps and parameters. Pearson correlation coefficient was calculated to measure the relationship strength between the log_10_ transformed tumor count data and each of the flexiVent and XV parameters, with results reported when *P*<0.05.

## References

[DMM052559C1] Asosingh, K., Frimel, M., Zlojutro, V., Grant, D., Stephens, O., Wenger, D., Fouras, A., DiFilippo, F. and Erzurum, S. (2022). Preclinical four-dimensional functional lung imaging and quantification of regional airflow: a new standard in lung function evaluation in murine models. *Am. J. Respir. Cell Mol. Biol.* 67, 423-429. 10.1165/rcmb.2022-0055MA 35687482 PMC9564925

[DMM052559C2] Bankhead, P., Loughrey, M. B., Fernández, J. A., Dombrowski, Y., McArt, D. G., Dunne, P. D., McQuaid, S., Gray, R. T., Murray, L. J., Coleman, H. G. et al. (2017). QuPath: open source software for digital pathology image analysis. *Sci. Rep.* 7, 16878. 10.1038/s41598-017-17204-5 29203879 PMC5715110

[DMM052559C3] Bray, F., Laversanne, M., Sung, H., Ferlay, J., Siegel, R. L., Soerjomataram, I. and Jemal, A. (2024). Global cancer statistics 2022: GLOBOCAN estimates of incidence and mortality worldwide for 36 cancers in 185 countries. *CA Cancer J. Clin.* 74, 229-263. 10.3322/caac.21834 38572751

[DMM052559C4] Cancer Genome Atlas Research Network (2014). Comprehensive molecular profiling of lung adenocarcinoma. *Nature* 511, 543-550. 10.1038/nature13385 25079552 PMC4231481

[DMM052559C5] Chevallier, M., Borgeaud, M., Addeo, A. and Friedlaender, A. (2021). Oncogenic driver mutations in non-small cell lung cancer: past, present and future. *World J. Clin. Oncol.* 12, 217-237. 10.5306/wjco.v12.i4.217 33959476 PMC8085514

[DMM052559C6] Devos, F. C., Maaske, A., Robichaud, A., Pollaris, L., Seys, S., Lopez, C. A., Verbeken, E., Tenbusch, M., Lories, R., Nemery, B. et al. (2017). Forced expiration measurements in mouse models of obstructive and restrictive lung diseases. *Respir. Res.* 18, 123. 10.1186/s12931-017-0610-1 28629359 PMC5477381

[DMM052559C7] Madero-Visbal, R. A., Colon, J. F., Hernandez, I. C., Limaye, A., Smith, J., Lee, C. M., Arlen, P. A., Herrera, L. and Baker, C. H. (2012). Bioluminescence imaging correlates with tumor progression in an orthotopic mouse model of lung cancer. *Surg. Oncol.* 21, 23-29. 10.1016/j.suronc.2010.07.008 20801643

[DMM052559C8] Muzumdar, M. D., Dorans, K. J., Chung, K. M., Robbins, R., Tammela, T., Gocheva, V., Li, C. M.-C. and Jacks, T. (2016). Clonal dynamics following p53 loss of heterozygosity in Kras-driven cancers. *Nat. Commun.* 7, 12685. 10.1038/ncomms12685 27585860 PMC5025814

[DMM052559C9] Nolte, P., Dullin, C., Svetlove, A., Brettmacher, M., Rußmann, C., Schilling, A. F., Alves, F. and Stock, B. (2022). Current approaches for image fusion of histological data with computed tomography and magnetic resonance imaging. *Radiol. Res. Pract.* 2022, 1-20. 10.1155/2022/6765895 PMC9668453 36408297

[DMM052559C10] Percie du Sert, N., Hurst, V., Ahluwalia, A., Alam, S., Avey, M. T., Baker, M., Browne, W. J., Clark, A., Cuthill, I. C., Dirnagl, U. et al. (2020). The ARRIVE guidelines 2.0: updated guidelines for reporting animal research. *PLoS Biol.* 18, e3000410. 10.1371/journal.pbio.3000410 32663219 PMC7360023

[DMM052559C11] Reyne, N., Cmielewski, P., McCarron, A., Smith, R., Schneider-Futschik, E., Eikelis, N., Pirakalathanan, P., Parsons, D. and Donnelley, M. (2024a). Effect of elexacaftor-tezacaftor-ivacaftor on nasal potential difference and lung function in Phe508del rats. *Front. Pharmacol.* 15, 1362325. 10.3389/fphar.2024.1362325 38545546 PMC10965794

[DMM052559C12] Reyne, N., Smith, R., Cmielewski, P., Eikelis, N., Lawrence, M., Louise, J., Pirakalathanan, P., Parsons, D. and Donnelley, M. (2024b). Assessment of respiratory mechanics and X-ray velocimetry functional imaging in two cystic fibrosis rat models. *Sci. Rep.* 14, 21646. 10.1038/s41598-024-71632-8 39284856 PMC11405763

[DMM052559C13] Reyne, N., Smith, R., Cmielewski, P., Eikelis, N., Nilsen, K., Louise, J., Duerr, J., Mall, M. A., Lawrence, M., Parsons, D. et al. (2025). functional lung imaging identifies peripheral ventilation changes in β-ENaC mice. *Respirology* 30, 335-345. 10.1111/resp.70009 39998270 PMC11965024

[DMM052559C14] Smith, R., Thomas, C., Nguyen, P., Badiei, A., Eikelis, N., Nilsen, K., Pirakalathanan, P., Parsons, D. and Donnelley, M. (2025). Visualising ventilation changes following endobronchial valve placement with x-ray velocimetry functional lung imaging. *Phys. Med. Biol.* 70, 135003. 10.1088/1361-6560/ade196 40472865

[DMM052559C15] Stahr, C. S., Samarage, C. R., Donnelley, M., Farrow, N., Morgan, K. S., Zosky, G., Boucher, R. C., Siu, K. K. W., Mall, M. A., Parsons, D. W. et al. (2016). Quantification of heterogeneity in lung disease with image-based pulmonary function testing. *Sci. Rep.* 6, 29438. 10.1038/srep29438 27461961 PMC4962033

[DMM052559C16] Tuveson, D. A., Shaw, A. T., Willis, N. A., Silver, D. P., Jackson, E. L., Chang, S., Mercer, K. L., Grochow, R., Hock, H., Crowley, D. et al. (2004). Endogenous oncogenic K-rasG12D stimulates proliferation and widespread neoplastic and developmental defects. *Cancer Cell* 5, 375-387. 10.1016/S1535-6108(04)00085-6 15093544

[DMM052559C17] Weeden, C. E., Gayevskiy, V., Marceaux, C., Batey, D., Tan, T., Yokote, K., Ribera, N. T., Clatch, A., Christo, S., Teh, C. E. et al. (2023). Early immune pressure initiated by tissue-resident memory T cells sculpts tumor evolution in non-small cell lung cancer. *Cancer Cell* 41, 837-852.e6. 10.1016/j.ccell.2023.03.019 37086716

[DMM052559C18] Zaw Thin, M., Moore, C., Snoeks, T., Kalber, T., Downward, J. and Behrens, A. (2022). Micro-CT acquisition and image processing to track and characterize pulmonary nodules in mice. *Nat. Protoc.* 18, 990-1015. 10.1038/s41596-022-00769-5 36494493

